# Long-term functional outcomes after unilateral versus bilateral decompressive craniectomy—a single center experience

**DOI:** 10.3389/fneur.2026.1826684

**Published:** 2026-07-03

**Authors:** D. Baldaranov, Ch. Großmann, K. Stangl, M. Kilic, S. Grubwinkler, R. A. Linker, F. Schlachetzki

**Affiliations:** 1 Department of Neurology, medbo Bezirksklinikum Regensburg, University of Regensburg, Regensburg, Germany; 2 Alzheimer Therapeutic Research Institute (ATRI), Keck School of Medicine of the University of Southern California, San Diego, CA, United States

**Keywords:** bilateral decompressive craniectomy, cranioplasty, outcomes, TBI, unilateral decompressive craniectomy

## Abstract

**Background:**

Early initiation of neurological rehabilitation following stroke or traumatic brain injury requires access to ventilation and comprehensive care at neurorehabilitation centers. The recovery after decompressive craniectomy (DC) is very heterogeneous and influenced by surgical laterality and complications. This study compared long-term outcomes after unilateral (UDC) versus bilateral DC (BDC).

**Methods:**

Patients admitted to our neurological rehabilitation center between 2000 and 2018 after BDC were matched by age, sex, and etiology to UDC. Clinical data included initial Glasgow Coma Scale, intracranial lesions, hospital admission time, complications, outcome, ventriculoperitoneal shunts and time to cranioplasty. Functional outcomes were assessed using the Extended Glasgow Outcome Scale (GOSE), Barthel Index (BI), and Early Rehabilitation Barthel Index (eBI) at discharge and follow-up. Follow-up interviews were conducted with 28 patients (15 UDC, 13 BDC) to evaluate GOSE, BI, quality of life, and home circumstances.

**Results:**

Fifty patients (mean age 28.2 ± 13 years; 28% female) were analyzed. Favorable outcomes occurred in 36% of UDC patients versus 16% of BDC patients, while unfavorable outcomes were more frequent in BDC (44% vs. 24%). UDC patients demonstrated significantly better eBI scores at discharge and follow-up (*p* = 0.043; *p* = 0.016) and superior GOSE outcomes (*p* = 0.011). Shorter hospitalization correlated with favorable outcomes in UDC (*p* = 0.012; *r* = −0.628). BDC patients experienced more neurological complications, which were associated with poorer outcomes (GOSE follow-up *p* = 0.019; *r* = −0.411; BI follow-up *p* = 0.022; *r* = 0.415).

**Conclusion:**

BDC is associated with poorer functional outcomes and higher complication rates compared to UDC. Further randomized studies are needed to confirm these findings.

## Introduction

Decompressive craniectomy (DC) is a well-established intervention for the management of critically elevated intracranial pressure (ICP) in conditions such as malignant cerebral infarction, traumatic brain injury, and intracerebral hemorrhage (1, 2). Depending on the extent and distribution of cerebral edema, DC may be performed as a unilateral or bilateral procedure (3). While unilateral decompressive craniectomy is typically used for focal pathology confined to one hemisphere, bilateral approaches—including bifrontal or bilateral frontotemporoparietal craniectomy—are reserved for diffuse cerebral swelling or multifocal injury. In addition, a distinction is made between primary and secondary DC. Primary DC is performed with the specific aim in cases where focal lesions are surgically accessible (e.g., evacuating hematomas). In most cases, this involves a unilateral frontotemporal craniectomy, although in certain situations it may also be performed as a bilateral temporal procedure (4). Secondary craniectomies are indicated in cases of diffusely increasing intracranial pressure and cerebral edema. A large proportion of secondary procedures are performed as bifrontal craniectomy (5, 6). This technique is used in situations such as generalized cerebral edema, bilateral frontal contusions, and intracranial hypertension that cannot be controlled with medical therapy.

Despite its widespread use, comparative data on outcomes between unilateral and bilateral DC remain limited. Differences in surgical extent, underlying pathology, and complication profiles may influence recovery and long-term outcomes, underscoring the need for further investigation (3).

Following DC, patients typically undergo cranioplasty (CP) to restore cranial integrity. Although often considered part of standard care, CP is associated with procedure-related complications that may affect neurological recovery and overall outcome (7). The interaction between initial surgical approach and subsequent complications remains insufficiently characterized.

The aim of this study is to present our single-center experience and to compare longitudinal outcomes and rehabilitation trajectories between patients undergoing bilateral DC and an age-, sex-, and etiology-matched cohort of patients after unilateral DC, and explores factors influencing these trajectories procedures.

## Methods

### Patients

All patients were treated at NRBKR between 2000 and 2018. The study included 25 patients who underwent bilateral craniectomy. Age at intervention ranged from 16 to 64 years, and all participants were adults at follow-up. A comparison group of 25 patients who underwent unilateral craniectomy was selected from the same center and matched to the bilateral cohort by age at surgery, sex, and etiology of increased intracranial pressure. The study protocol was conducted in agreement with the guidelines of the Declaration of Helsinki and was approved by the Ethics Committee of the University of Regensburg (Nr. 18-1040-101). All patients or their legal representatives provided written informed consent prior to enrolment. Following review of the medical records, patients were contacted and asked to complete a Follow Up questionnaire.

Initial retrospective data collection was based on hospital medical records and included: demographics, in-hospital course, initial Glasgow Coma Scale (GCS), time from hospitalization to DC, as well as the intracranial lesions characteristics (8). Lesions were very heterogenous, including epidural and subdural hematoma, subarachnoid hemorrhage, cerebral contusions (with and without bleeding), and diffuse axonal trauma. Lesions were classified as unilateral and bilateral lesions.

The selected time points were at admission to our Neurorehabilitation care unit (NRU), time of transition to phase B (early post-acute phase neurorehabilitation) as defined else were, at discharge from the NRU, and at Follow Up (9).

### Outcome measures (including Follow Up)

Patient outcomes were assessed using three validated instruments:

Barthel Index (BI) is widely used to evaluate the ability to perform the activities of daily living (ADL) (10). Patients can score from 0 to 100 points, with 100 indicating full independence (continence, self-feeding, dressing, mobility, bathing, walking at least one block, and stair climbing). A score >75 was defined as a favorable outcome (11, 12). BI does not have a separate score to indicate patients that passed away. For this reason, in the Follow Up patients who did not survive were counted as missing in the BI.

The assessment on the Extended Glasgow Outcome Scale (GOSE) was based on discharge reports (13). Patients can score from 1 to 8, where 1 indicates death and 8 represents complete recovery with normal life and work capacity, without neurological or psychological deficits. Outcomes were dichotomized into unfavorable (GOSE 1–4) and favorable (GOSE 5–8), consistent with previous studies (14, 15).

The Early Rehabilitation Barthel Index (ErBI) assesses functional status during early rehabilitation (16). It subtracts negative points for conditions requiring intensive medical monitoring (e.g., tracheostoma care, intermittent ventilation, confusion, behavioral disturbances, swallowing disorders, severe communication deficits) from the positive BI score. Positive score indicates favorable outcome.

At follow-up, patients completed a questionnaire including GOSE and BI. Additionally, two items adapted from the Quality-of-Life Scale were added: “It’s difficult for me to concentrate” (17), “My personality has changed after the event.” These two additional items addressing concentration difficulties and perceived personality changes were included as exploratory, to capture cognitive and psychosocial sequelae not reflected by standard outcome measures; these items were not formally validated and were analyzed descriptively.

### Complications

Complications were categorized as neurological and non-neurological. Neurological complications included post-interventional hemorrhage, cerebral infarctions, epilepsy and vasospasm. Additional neurological issues following DC were alterations of cerebrospinal fluid dynamics such as subdural hygroma or a persisting hydrocephalus. The latter may require ventriculoperitoneal shunt (VPS) which itself carries a risk of infection. To obtain more reliable data on improvement of neurogenic dysphagia we documented the timing of tracheostomy indication. Non-neurological complications, e.g., urinary tract or tracheostomy/craniectomy wound infections.

### VP shunt/cranioplasty

Hydrocephalus is a common complication after DC which leads to a range of neurological symptoms (18). VPS placement was performed in such patients. It was of interest when VPS was performed, before or after cranioplasty (CP). The interval between DC and CP was recorded, as well as the complications associated with CP (e.g., wound infections, hemorrhage, epilepsy, revision surgery or a hydrocephalus due to CP).

### Statistics

All data was collected and processed via SPSS (Version 25). Parametric data were presented as the mean ± SD, the quantity of participants with available data (*n*) and the range.

All statistical analyses were conducted using two-sided tests. *p*-values ≤ 0.05 were considered statistically significant, ≤ 0.01 highly significant, and < 0.001 extremely significant. Normality was assessed using graphical methods and the Kolmogorov–Smirnov test. As none of the variables followed a normal distribution due to the small sample size, nonparametric methods were applied.

Specifically, rank-sum tests were used, as they require fewer assumptions than parametric tests such as the *t*-test and are independent of underlying distributional forms. The Mann–Whitney U test was primarily employed to compare two independent groups (bilateral vs. unilateral patients). If *p* was <0.05, the result was statistically significant (*). If *p* ≤ 0.01 it was marked with two stars (**), *p* < 0.001 was marked with three stars (***). The null hypothesis was formulated and retained or rejected based on the test results. Unequal sample sizes were permitted for the Mann–Whitney U test. Correlation analysis was performed after Kendall (Kendall-Tau-b). A correlation coefficient >0.5 was evaluated as very high.

## Results

### Baseline characteristics

During the retrospective study period (2000–2018), all patients were treated at the Neurologische Rehabilitationsklinik am Bezirksklinikum Regensburg (NRBKR), a comprehensive neurorehabilitation center covering treatment phases B–D and including an in-house intensive care unit. Within this period, 25 patients (7 female) were admitted for neurorehabilitation after bilateral decompressive craniectomy (BDC) and prior to cranioplasty (CP). The mean age at admission was 28 years (median 23; interquartile range 13). From all patients admitted within the same timeframe after unilateral decompressive craniectomy, 25 were selected and matched for age, sex, and etiology as a comparison group. Etiology was classified only as traumatic brain injury (TBI) or spontaneous subarachnoid hemorrhage (SAH) without a traumatic cause. Patients who underwent unilateral craniectomy following infarction or isolated intracerebral hemorrhage were not included in the comparison group. Baseline characteristics of both groups are summarized in Table 1.

**Table 1:** Patients demographics.

Summary statistic		BDC	UDC	*p*-value
Age - years	Average (Median)	28.0 (23)	28.4 (20)	0.915^a^
	Interquartile range	13	17	
Female sex – no. (%)		7 (28)	7 (28)	1.000^a^
GCS	Average (Median)	4.21 (4.00)	4.59 (3.00)	0.862
	Interquartile range	3	4	
Time from hospitalization to DC - days		*n* = 25	*n* = 25	0.019*
	Average (Median)	3.6 (2)	1.5 (0)	
	Interquartile range	6	1	
Total duration time at NRU - days		*n* = 24	*n* = 24	0.317
	Average (Median)	261 (198)	186 (166)	
	Interquartile range	203	208	
Intracranial lesions				
	Bilateral intracranial lesions	20	5	
	Epidural hematoma	4	6	
	Subdural hematoma	10	17	
	SAH – traumatic	12	9	
	SAH – spontanous^a^	5	5	
	Traumatic hemorrhage	11	6	
	Diffuse axonal trauma	4	6	
	Combined intracranial lesions	18	15	

^a^Matched values.

At admission the GCS was numerically higher in the UDC (4.6 IR 4 vs. 4.21 IR3; *p* = 0.862). Craniectomy was performed in 40 patients with traumatic brain injury (TBI) and in 10 patients with spontaneous subarachnoid hemorrhage (SAH). In the bilateral craniectomy cohort, 20 patients (80%) had intracranial injuries involving both hemispheres, compared with 5 patients (20%) in the unilateral cohort (*p* = 0.001). Combined intracranial lesions were present in 18 patients (72%) in the bilateral cohort and 15 patients (60%) in the unilateral cohort. Among patients who underwent bilateral craniectomy, two (8%) had primary procedures, 17 (68%) secondary procedures, and nine (36%) staged bilateral craniectomies. In the comparison group of patients with unilateral craniectomy, 18 (72%) underwent primary and seven (28%) secondary procedures. The intracranial lesions are described in detail in Table 1.

The interval between hospitalization and the first DC was significantly longer for the BDC: (3.6 IR6 days) as for the UDC (1.5IR1 days; *p* = 0.019*).

**Figure 1:**
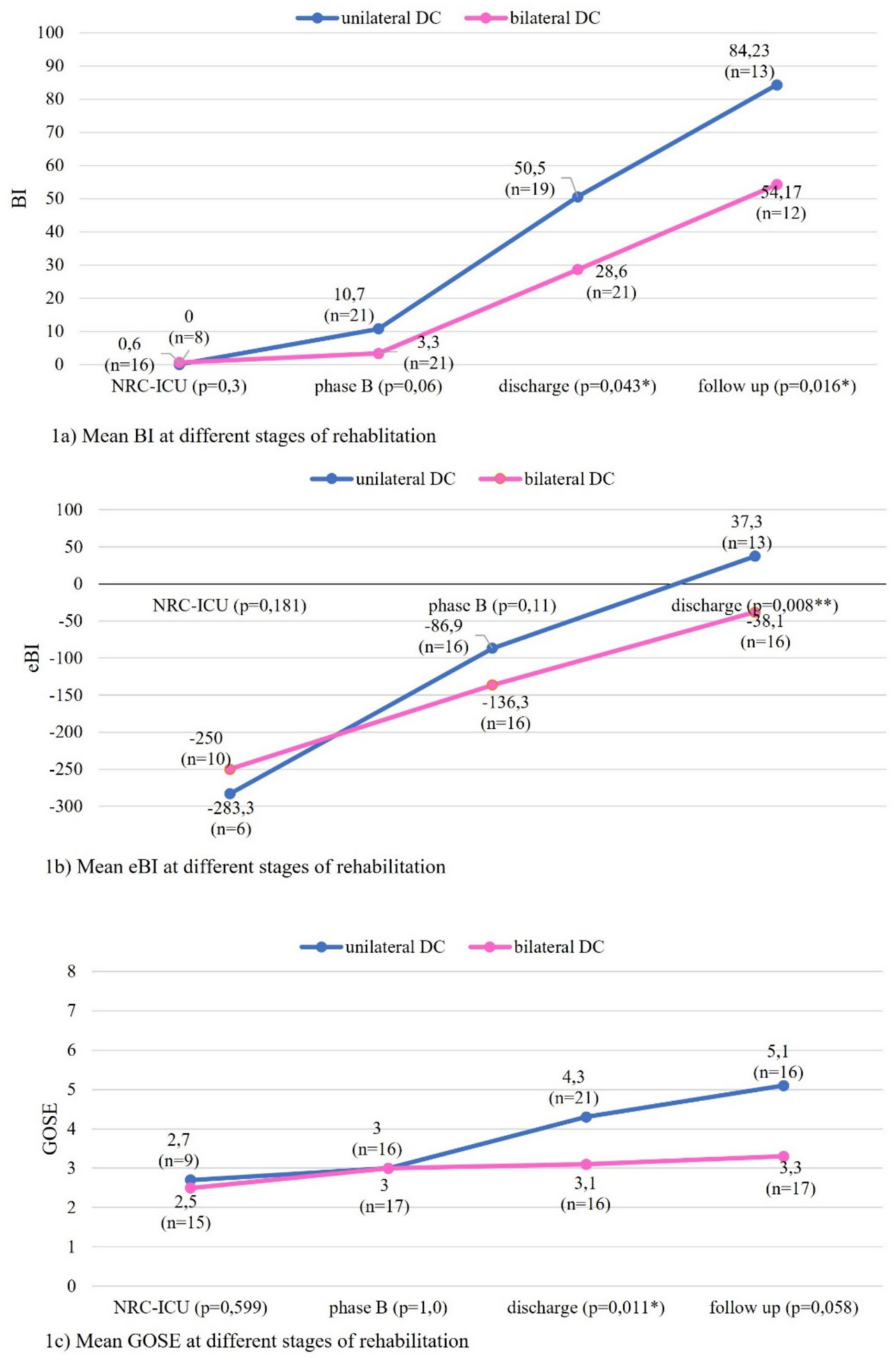
**(a)** Mean BI at the different timepoints; **(b)** mean eBI at the different timepoints; **(c)** mean GOSE at the different timepoints.

The neurological rehabilitation began for 27 patients (17 BDC and 10 UDC) with weaning from artificial ventilation at the intensive care unit (ICU) of the of our neurorehabilitation care unit (NRU). At this timepoint BI was similar between groups (BDC: 0.63 ± 1.71, *n* = 16, range 0–5; UDC: 0 ± 0, *n* = 8, range 0–0; *p* = 0.3) as well as the eBI (BDC: −250 ± 40.83, *n* = 10, range −325 to −200; UDC: −283.33 ± 49.16, *n* = 6, range −325 to −225; *p* = 0.181) (Figure 1).

### Outcome

BI, eBI, and GOSE showed a significant difference between the groups at discharge from NRU with *p*-values of 0.043**, 0.008**, 0.011*. The longitudinal improvement of our participant is depicted with these assessments in Figure 1.

### Follow Up

The Follow Up reached 32 patients and 26 of them (12 BDC and 14 UDC) answered the questionnaire. At this point 4 BDC and 2 UDC treated patients already passed away. 2 BDC lived in a nursing home. 5 BDC and 3 UDC were receiving professional nursing service at home.

The period between discharge from the NRU and the Follow Up was very heterogenous with a range between 3 months and 16 years. The mean period was 5.4 ± 5.05 years in BDC and 7.3 ± 4.83 years in UDC. The BDC (*n* = 12, range 0–100) reached a BI of 54.17 ± 42.20 where the UDC (*n* = 13, range 15–100) 84.23 ± 29.36 (*p* = 0.016*). At this last timepoint of the study a favorable outcome was reached by 5 (20%) BDC and by 10 (40%) UDC patients on GOSE and 6 (24%) and 10 (40%) on BI, respectively, (Table 2). Mean GOSE was 3.29 ± 2.66 (*n* = 17, range 1–8) for the BDC and 5.13 ± 2.52 (*n* = 16, range 1–8) for the UDC (*p* = 0.058).

**Table 2:** Favorable and unfavorable outcome at Follow Up; (A) GOSE; (B) BI.

(A) Favorable and unfavorable outcome (GOSE) at Follow Up		
GOSE	UDC	BDC
Favorable outcome (GOSE 5–8)	10 (40.0%)	5 (20.0%)
Unfavorable outcome (GOSE 1–4)	6 (24.0%)	12 (48.0%)
Total of patients reached at *Follow Up*	16 (64.0%)	17 (68.0%)

(B) Favorable and unfavorable Outcome (BI) at Follow Up		
Barthel Index	UDC	BDC
Favorable outcome (BI >85)	10 (40.0%)	6 (24.0%)
Unfavorable outcome (BI <85)	3 (12.0%)	6 (24.0%)
Total of patients reached at *Follow Up*	13 (52.0%)	12 (48.0%)

**Figure 2:**
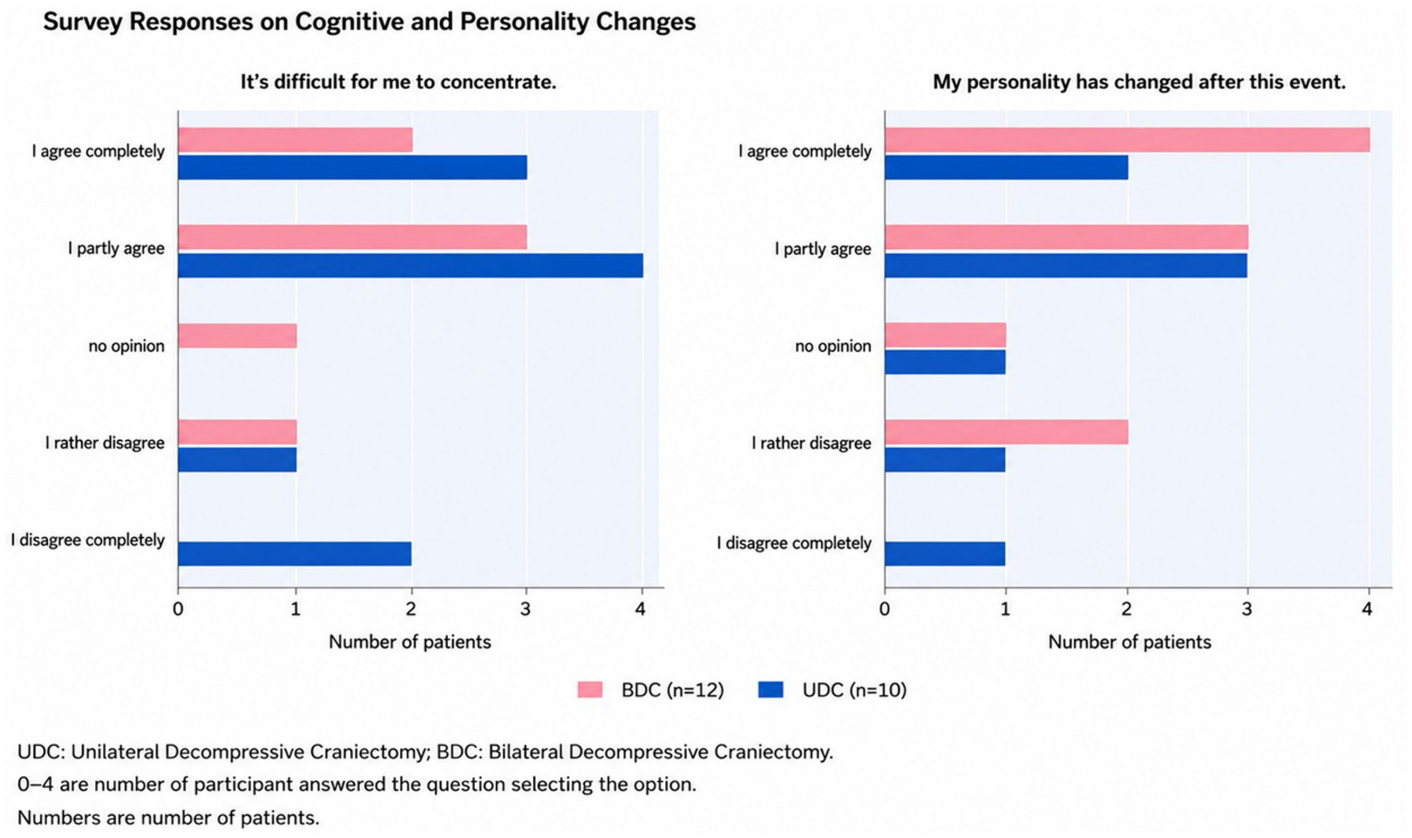
Survey responses on cognitive and personality changes following the event, comparing BDC (*n* = 12) and UDC (*n* = 10). **(a)** Displays responses to the statement “It’s difficult for me to concentrate,” showing higher overall agreement among UDC participants. **(b)** Presents responses to “My personality has changed after this event,” with stronger endorsement observed in the BDC group. Responses are distributed across five categories ranging from “I disagree completely” to “I agree completely”.

The survey results indicate notable differences between the BDC (*n* = 12) and UDC (*n* = 10) groups in reported cognitive and personality changes following the event. Regarding concentration difficulties (2a), a majority of UDC participants endorsed impairment, with 3 respondents (30%) indicating “I agree completely” and 4 (40%) “I partly agree,” meaning that 70% reported at least some difficulty concentrating. In contrast, BDC responses were more distributed: 2 participants (≈17%) “agreed completely” and 3 (25%) “partly agreed,” while a substantial proportion—approximately 5 respondents (≈42%)—“disagreed completely.” This suggests that concentration problems were more consistently reported within the UDC group. For perceived personality change (Item 2b), the pattern was reversed. In the BDC group, 4 participants (≈33%) “agreed completely” and 3 (25%) “partly agreed,” indicating that over half (≈58%) perceived some degree of personality change. Among UDC respondents, 2 participants (20%) “agreed completely” and 3 (30%) “partly agreed,” while several expressed disagreement. Overall, these findings suggest that cognitive difficulties were more prominent in the UDC group, whereas perceived personality changes were more strongly endorsed in the BDC group, highlighting differential patterns of post-event impact between the two groups (Figure 2).

### Complications

The most common complication was consecutive development of hydrocephalus and this occurred in 17 patients in the BDC cohort compared to 8 patients in the UDC, representing the largest difference between groups. Hygroma and VPS revision were more common in BDC patients, while secondary brain infarction and vasospasm were reported only in the unilateral cohort. Shunt infections and post-interventional bleeding were present in both groups but occurred more often in BDC patients. In addition, epilepsy and aspiration pneumonia were observed in both cohorts, with slightly more BDC patients affected (12 vs. 8 cases for epilepsy; 8 cases each for aspiration pneumonia).

Lower number of neurological complications correlated with high GOSE and BI at Follow Up [Kendall-Tau-b; *p*(GOSE) = 0.019; *p*(BI) = 0.022]. The tracheostomy tube remained for a mean of 110.32 ± 124.87 days (*n* = 19, range 15–491 days) in the BDC and for a mean of 80.56 ± 66.38 days (*n* = 18, range 5–193 days) in the UDC (*p* = 0.564). A high BI at discharge form the rehabilitation clinic correlated with short period of use of tracheostomy tube (Kendall-Tau-b; *p*(BI) = 0.008**, *r* = −0.463) as well as a high BI and high GOSE at Follow Up (Kendall-Taub-b; *p*(GOSE) = 0.020*, *r*(GOSE) = −0.542; *p*(BI) = 0.017*, *r*(BI) = −0.571).

**Figure 3:**
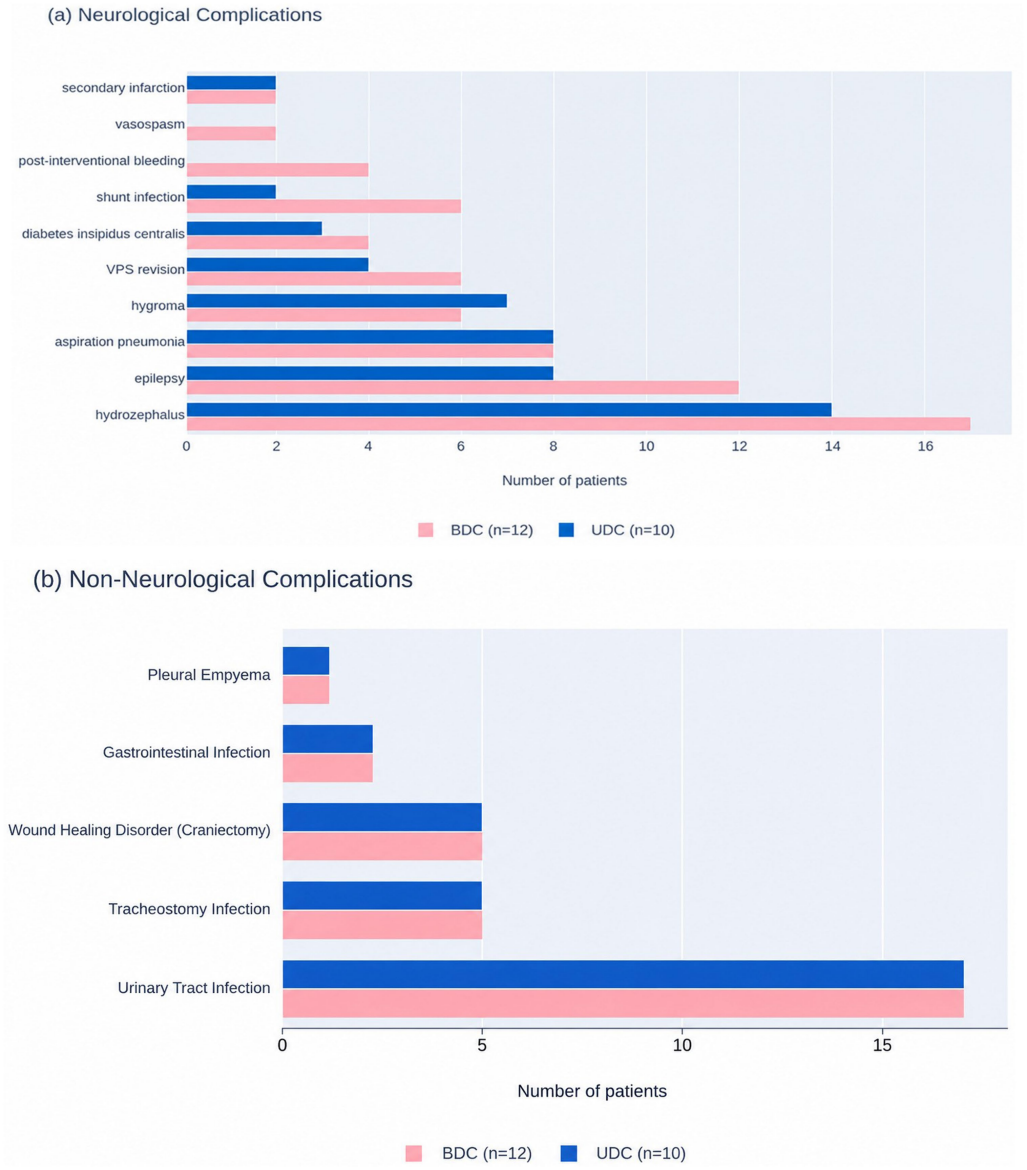
The occurrence of complications in both cohorts by number of patients developing such. **(a)** neurological complications; **(b)** non-neurological complications. BDC, Bilateral decompressive craniectomy; UDC, unilateral decompressive craniectomy.

In our study 4 (16%) BDC and 2 (8%) UDC treated patients did not experience any neurological complications during the study. Most of patients did not develop any non-neurological complications during their stay at the NRU (9 (36%) BDC, 14 (56%) UDC). However, 4 (16%) BDC and 5 (20%) UDC recovered after more than one non-neurological complication (Figure 3).

### Ventriculoperitoneal shunts

VPS for treatment of postinterventional hydrocephalus after DC was indicated more frequently in the BDC group [17 BDC (68%) vs. 8 (32%) UDC (*p* = 0.012)]. The intervention took place between 19 to 327 days after the patient’s DC [mean 120 ± 88 days (*n* = 17) BDC vs.71 ± 67 days (*n* = 8) UDC (*p* = 0.23)].

### Cranioplasty

**Figure 4:**
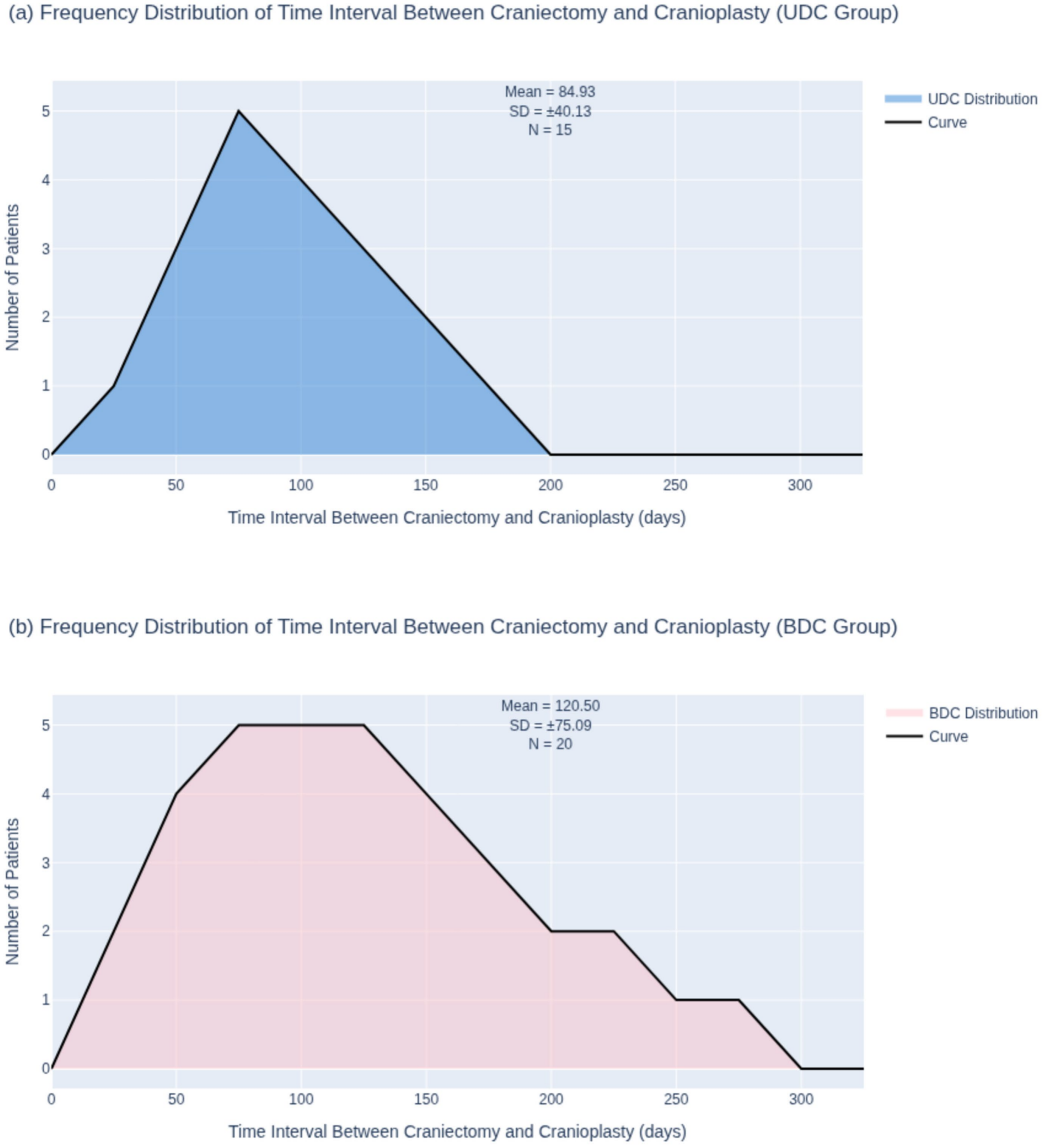
Frequency distribution of time interval (days) between craniectomy and cranioplasty: **(a)** UDC Group (*N* = 15). The mean interval was 84.93 days (SD = 40.13), showing variability in timing across patients. **(b)** BDC Group (*N* = 20). The mean interval was 120.50 days (SD = 75.09), indicating greater variability compared to the UDC group. The difference between groups was not statistically significant (*p* = 0.202).

Among the 50 patients analyzed, cranioplasty details were available for 20 BDC and 15 UDC patients (70%). The mean interval between craniectomy and cranioplasty was 84.93 ± 40.13 days in the UDC group (*N* = 15) and 120.50 ± 75.09 days in the BDC group (*N* = 20). The BDC group demonstrated a longer and more variable interval compared to the UDC group (Figure 4).

**Figure 5:**
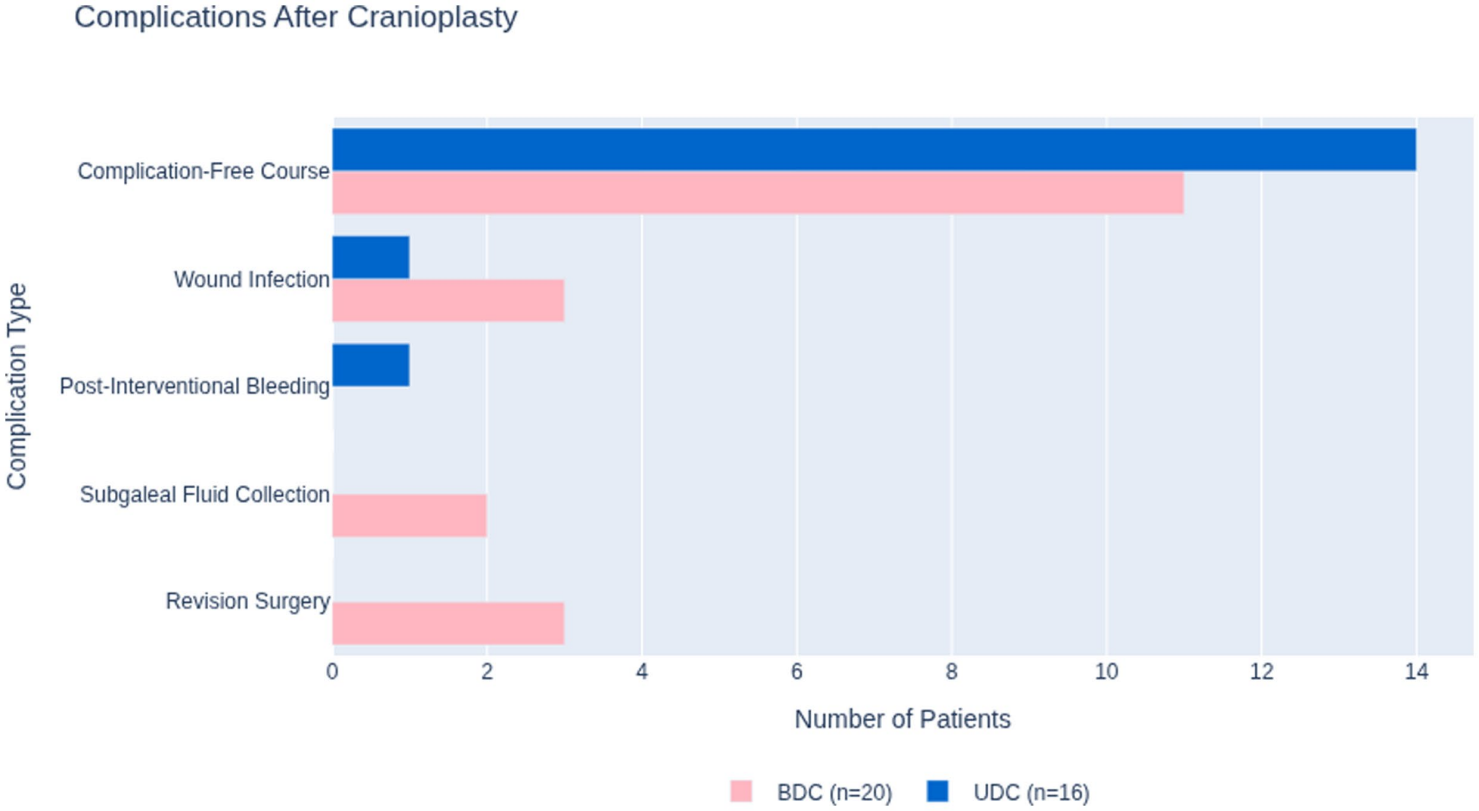
Complications after cranioplasty. BDC, Bilateral decompressive craniectomy; UDC, unilateral decompressive craniectomy.

Of the 35 cranioplasties recorded (20 BDC, 15 UDC), 25 procedures were complication-free (11 BDC;14 UDC; 29%). Four patients developed wound infections (3 BDC; 1 UDC), and one UDC patient experienced post-interventional bleeding in the form of epidural hematoma. Subgaleal fluid collection occurred in two BDC patients, and three BDC patients required revision surgery. Post-cranioplasty, one UDC and five BDC patients developed secondary cerebrospinal fluid drainage disorders requiring VP shunt implantation (Figure 5).

## Discussion

In this study, we compared long-term outcomes and rehabilitation trajectories in patients undergoing decompressive craniectomy (DC) and subsequent cranioplasty (CP). Patients were stratified into two groups: those who underwent bilateral decompressive craniectomy (BDC) and an age-, sex-, and etiology-matched cohort treated with unilateral decompressive craniectomy (UDC). A total of 50 patients (25 per group) were included in the study.

The mean in-hospital stay was 261.13 ± 241.02 days for the BDC group and 185.83 ± 134.98 days for the UDC group. The mean follow-up period after discharge from the neurorehabilitation unit (NRU) was 5.4 ± 5.05 years for BDC patients and 7.3 ± 4.83 years for UDC patients.

Our analysis showed that patients treated with UDC achieved significantly better functional recovery compared to those undergoing BDC. This difference was consistent across multiple outcome measures, including the Barthel Index (BI), extended Barthel Index (eBI), and Glasgow Outcome Scale Extended (GOSE). At both discharge and follow-up, UDC patients demonstrated higher BI scores, indicating greater independence in activities of daily living, and lower disability levels as reflected by eBI. Likewise, GOSE scores were higher in the unilateral group, suggesting superior overall functional outcomes.

Several factors may explain these findings. Patients in the BDC group experienced a more severe initial clinical course. Some underwent sequential craniectomy, and the time from hospital admission to decompressive surgery was, on average, 2 days longer than in UDC patients, potentially resulting in prolonged exposure to elevated intracranial pressure. Although BI scores at NRU admission were comparable between groups, a higher proportion of BDC patients required ventilator weaning prior to rehabilitation.

An age-dependent effect on outcome was not confirmed. In the BDC cohort, patients with favorable GOSE outcomes were, on average, older than those with unfavorable outcomes. In the UDC group, a non-significant trend toward better outcomes in younger patients was observed. Overall, these findings suggest that advanced age does not necessarily preclude favorable recovery.

The initial Glasgow Coma Scale (GCS) demonstrated limited prognostic value. A positive association with discharge outcome was observed only in the BDC group, while no significant correlation was found at follow-up in either cohort. No significant intergroup differences were identified.

More than half of the patients in both groups presented with combined intracranial lesions, reflecting the heterogeneity of severe traumatic brain injury (TBI). Notably, bilateral injuries were also observed in some patients treated with unilateral craniectomy and vice versa. This likely reflects differences in surgical strategy: primary DC is often performed unilaterally to address focal lesions, whereas diffuse intracranial hypertension may require secondary, frequently bilateral, decompression.

Rates of neurological complications, ventriculoperitoneal shunt placement, and time to cranioplasty all favored the UDC group. In summary, these early and cumulative factors in patients undergoing BDC contribute to a less favorable rehabilitation trajectory and a higher degree of long-term disability. Our findings align closely with prior research underscoring the prognostic importance of surgical extent in severe traumatic brain injury (TBI). Zhao et al. evaluated 151 patients who underwent decompressive craniectomy (DC) for severe TBI, including epidural hematoma, subdural hematoma, intraparenchymal hemorrhage, brain contusion, and brain laceration (19). Their cohort (mean age 55.2 years, 27% female, mean admission GCS ~ 9) demonstrated a high rate of unfavorable outcomes (62%, defined as mRS 4–5). Importantly, patients who required a delayed contralateral decompressive procedure experienced particularly poor functional outcomes. An important finding was that lower admission GCS scores, higher intracranial pressure (ICP) burden are factors strongly correlated with greater disability and poorer functional outcomes. Similarly, large, randomized trials and population-based studies—including DECRA, RESCUEicp, and the work by Honeybul et al.—have demonstrated that although decompressive craniectomy (DC), particularly when performed bilaterally, may reduce mortality, it does not necessarily translate into favorable functional recovery (20–22).

The DECRA trial, conducted in a relatively young cohort (mean age ~34 years; admission GCS ~ 5), reported worse functional outcomes at 6 months following early DC despite effective intracranial pressure control (20). Moreover, patients who underwent bilateral decompressive craniectomy were significantly more likely to experience an unfavorable outcome (Glasgow Outcome Scale [GOS] ≥ 3) compared with those undergoing unilateral procedures (odds ratio 4.42; 95% CI 1.16–16.81; *p* = 0.029), even after adjusting for surgical timing, mechanism of injury, and predicted risk.

Similarly, the RESCUEicp trial demonstrated improved survival at 24 months but with a substantial proportion of patients remaining severely disabled, underscoring the persistent tension between life-saving intervention and long-term functional independence (21). These findings mirror our results, where more extensive surgical intervention was associated with survival benefit but not proportional gains in functional recovery.

In a population-based study, Honeybul et al. evaluated outcomes at 6 and 18 months and reported high rates of unfavorable outcome in both unilateral (55% at 6 months; 41% at 18 months) and bilateral DC (45% at 6 months; 36% at 18 months), as measured by the GOS (22) Notably, functional improvement was observed between the two time points, a trajectory that is consistent with the delayed recovery pattern observed in our cohort. Their study population was younger (mean age 33.1 ± 15 years), had a broader range of injury mechanisms (including assault and blast injuries), and presented with slightly better initial neurological status. Despite these demographic and injury-pattern differences, the overall pattern of persistent disability following more extensive decompression reinforces our findings that surgical magnitude alone does not ensure favorable long-term neurological recovery and may instead reflect underlying injury severity.

However, in our study we followed up our participants also after cranioplasty. Previous rehabilitation-focused studies confirm that improvements on functional scales, e.g., GOSE are modest and strongly influenced by initial injury severity and timing of cranioplasty, rather than DC type alone (23). Early cranioplasty may enhance cognitive and motor recovery, but even then BDC patients remain at higher risk for persistent deficits. With an average time to CP of 120d for the BDC and 85d for UDC our participants are more likely to be in the early group as defend by De Cola et al. (24) which may influence the functional and cognitive recovery while enhancing cerebral blood flow. Yet, bilateral patients remain disadvantaged due to diffuse injury burden.

In addition, in our study the participants responses on the QoL questions are hinting that individual subjective cognitive and psychosocial challenges persist long-term in both cohorts and are, suggesting a distinct patterns of impact between the two groups. A majority of UDC participants (70%) reported at least some difficulty concentrating, indicating that cognitive functioning may have been more strongly affected in this group. In contrast, BDC participants more frequently endorsed personality changes (approximately 58%), with a higher proportion selecting “agree completely,” suggesting a more pronounced perceived shift in self-concept or personal characteristics. Overall, the results may allow the assumption that UDC participants experienced stronger cognitive effects, whereas BDC participants reported more substantial personality-related changes following the event. Importantly, thus classified as “favourable” by BI or GOSE participants often report persistent concentration difficulties and personality changes, stressing out the limitations of functional scales in capturing long-term psychosocial sequelae. Much more, these results are providing preliminary insights of the magnitude of influence on all day life the accidents, surgeries and long rehabilitation processes are having on the patient’s causing limitation in their functioning, social and family life, very often in young age. Integrating these patient-reported outcomes emphasizes the need for individualized rehabilitation strategies and closer monitoring of bilateral DC patients, regarding cognitive rehabilitation.

### Strength and limitation

The present study is characterized by several strengths that enhance the relevance of the results. The study allowed for a comparison between groups, with careful matching of key variables age, sex and etiology. The relatively long follow-up period, enabling the assessment of long-term outcomes rather than short-term effects alone. Additionally, the use of standardized and widely validated outcome measures (BI, GOSE) strengthens the reliability and comparability of the results. Another important strength is the comprehensive data collection across multiple time points (e.g., admission, discharge, follow-up), which allows for a detailed evaluation of rehabilitation trajectories. Although caution is warranted in interpretation, the study provides clinically relevant insights and incorporates aspects of cognitive and psychosocial sequelae.

This study has several limitations that must be acknowledged. Its retrospective design and small sample size are limiting the generalizability of the findings. Although we matched cohorts for age, sex, and ethology of intracranial hypertension, residual confounding factors such as injury severity, comorbidities, and variations in acute care may have influenced outcomes. In addition, selection bias is present, as only patients who survived the acute phase and were admitted to our neurorehabilitation unit were included potentially skewing functional outcome comparisons. Also, the heterogeneity of intracranial lesions and the wide range of follow-up durations (3 months to 16 years) complicate interpretation of long-term recovery trajectories. Finally, cranioplasty timing and technique were not standardized, which may have influenced recovery independently of DC type. In addition, the observed patterns—differential reporting of cognitive versus personality changes between UDC and BDC groups—are consistent with known effects of focal versus diffuse brain injury, supporting the face validity and clinical plausibility of these measures. Future studies should incorporate fully validated neuropsychological and quality-of-life instruments to better characterize these domains in a standardized manner.

A prospective, multicentre study with larger cohort and standardized rehabilitation protocol may bring more insights on the mechanisms underlying poorer outcomes after bilateral DC and to identify modifiable factors that could improve long-term recovery.

### Summary

From a clinical perspective, these findings have several implications. First, the indication for bilateral decompression should be carefully considered, as it is associated with a substantially higher burden of complications and poorer functional recovery. Second, early identification and management of complications—particularly hydrocephalus—are critical in improving outcomes in BDC patients. Third, rehabilitation strategies should be individualized: UDC patients may benefit more from targeted cognitive rehabilitation, whereas BDC patients may require broader neuropsychological and behavioral support. Finally, optimizing the timing of cranioplasty may represent a modifiable factor to enhance recovery.

## Data Availability

The original contributions presented in the study are included in the article/supplementary material, further inquiries can be directed to the corresponding author.
